# Supplementary material for: Discovery and application of insertion-deletion (INDEL) polymorphisms for QTL mapping of early life-history traits in Atlantic salmon
Source: BMC Genomics. 2010 Mar 8;11:156. doi: 10.1186/1471-2164-11-156 (PMC2838853; doi:10.1186/1471-2164-11-156)
Supplement: Additional file 2 — Information on developed 76 locus single-run INDEL panel in Atlantic salmon. Information on fluorescence labeling, primer concentrations, PCR pooling and links to alignments, INDEL motifs and GENESCAN (Burge and Karlin 1997) predictions of genes/exons are available in html format. [file 1471-2164-11-156-S2.ZIP › Additionalfile2/table20056.html]

Contig Alignment

```
Base	A B C D   
1	. . C .    
2	. . C .    
3	. . C .    
4	. . C .    
5	. . C .    
6	. . G .    
7	. . T .    
8	. . A .    
9	. . A .    
10	. . G .    
11	. . A .    
12	. . G .    
13	. . A .    
14	. . C .    
15	. . G .    
16	. . A .    
17	. . T .    
18	. . T .    
19	. . A .    
20	. . C .    
21	. . A .    
22	. . A .    
23	. . A .    
24	. . G .    
25	. . C .    
26	. . C .    
27	. . C .    
28	. . A .    
29	. . T .    
30	. . G .    
31	. . G .    
32	. . G .    
33	. . A .    
34	. . T .    
35	. . G .    
36	. . G .    
37	. . G .    
38	. . T .    
39	. . G .    
40	. . G .    
41	. . T .    
42	. . G .    
43	. . A .    
44	. . T .    
45	. . G .    
46	. . C .    
47	. . T .    
48	. . C .    
49	. . C .    
50	. . T .    
51	. . G .    
52	. . G .    
53	. . T .    
54	. . G .    
55	. . G .    
56	. . A .    
57	. . A .    
58	. . T .    
59	. . C .    
60	. . A .    
61	. . A .    
62	. . A .    
63	. . G .    
64	. . G .    
65	. . G .    
66	. . A .    
67	. . C .    
68	. . C .    
69	. . A .    
70	. . G .    
71	. . T .    
72	. . T .    
73	. . T .    
74	. . G .    
75	. . G .    
76	. . A .    
77	. . T .    
78	. . A .    
79	. . T .    
80	. . G .    
81	. . T .    
82	. . T .    
83	. . C .    
84	. . T .    
85	. . C .    
86	. . C .    
87	. . A .    
88	. . G .    
89	. . A .    
90	. . G .    
91	. . A .    
92	. . A .    
93	. . G .    
94	. . G .    
95	. . A .    
96	. . G .    
97	. . A .    
98	. . A .    
99	. . G .    
100	. . G .    
101	. . G .    
102	. . C .    
103	. . A .    
104	. . A .    
105	. . A .    
106	. . A .    
107	. . C .    
108	. . A .    
109	. . G .    
110	. . C .    
111	. . A .    
112	. . C .    
113	. . G .    
114	. . G .    
115	. . A .    
116	. . G .    
117	. . A .    
118	. . A .    
119	. . G .    
120	. . T .    
121	. . T .    
122	. C C .    
123	. A A .    
124	. G G .    
125	. C C .    
126	. A A .    
127	. A A .    
128	. A A .    
129	. G G .    
130	. T T .    
131	. G G .    
132	. G G .    
133	. G G .    
134	. A A .    
135	. C C .    
136	. G G .    
137	. C C .    
138	. A A .    
139	. A A .    
140	. C C .    
141	. A A .    
142	. G G .    
143	. T T .    
144	. C C .    
145	. T T .    
146	. G G .    
147	. G G .    
148	. A A .    
149	. C C .    
150	. A A .    
151	. T T .    
152	. T T .    
153	. T T .    
154	. T T .    
155	. C C .    
156	. T T .    
157	. C C .    
158	. T T .    
159	. C C .    
160	. C C .    
161	. A A .    
162	. G G .    
163	. T T .    
164	. C C .    
165	. A A .    
166	. G G .    
167	. A A .    
168	. G G .    
169	. A A .    
170	. G G .    
171	. G G .    
172	. A A .    
173	. T T .    
174	. T T .    
175	. A A A    
176	. C C C    
177	. A A A    
178	. A A A    
179	. A A A    
180	. T T T    
181	. C C C    
182	. C C C    
183	. C C C    
184	. A A A    
185	. T T T    
186	. G G G    
187	. G G G    
188	. A A A    
189	. A A A    
190	. T T T    
191	. G G G    
192	. G - -  1	 indel  
193	. A A A    
194	. C C C    
195	. C C C    
196	. G G G    
197	. C C C    
198	. A A A    
199	. G G G    
200	. A A A    
201	. T T T    
202	. G G G    
203	. T T T    
204	. A A A    
205	. A A A    
206	. C C C    
207	. A A A    
208	. A A A    
209	. G G G    
210	. T T T    
211	. G G G    
212	. G G G    
213	. G G G    
214	. A A A    
215	. A A A    
216	. C C C    
217	. A A A    
218	. A A A    
219	. A A A    
220	. G G G    
221	. G G G    
222	. A A A    
223	. A A A    
224	. A A A    
225	. C C C    
226	. A A A    
227	. A A A    
228	. T T T    
229	. C C C    
230	. T T T    
231	. G G G    
232	. G G G    
233	. A A A    
234	. C C C    
235	. T T T    
236	. T T T    
237	. C C C    
238	. C C C    
239	. T T T    
240	. C C C    
241	. C C C    
242	. C C C    
243	. T T T    
244	. T T T    
245	. C C C    
246	. C C C    
247	. A A A    
248	. A A A    
249	. C C C    
250	. T T T    
251	. C C C    
252	. T T T    
253	. G G G    
254	. G G G    
255	. T T T    
256	. G G G    
257	. G G G    
258	. A A A    
259	. C C C    
260	. C C C    
261	. C C C    
262	. C C C    
263	. C C C    
264	. A A A    
265	. C C C    
266	. A A A    
267	. C C C    
268	. C C C    
269	. G G G    
270	. C C C    
271	. A A A    
272	. A A A    
273	. G G G    
274	. A A A    
275	. C C C    
276	. T T T    
277	. C C C    
278	. C C C    
279	. C C C    
280	. C C C    
281	. T T T    
282	. G G G    
283	. G G G    
284	. G G G    
285	. C C C    
286	. A A A    
287	. C C C    
288	. A A A    
289	. C C C    
290	. C C C    
291	. T T T    
292	. G G G    
293	. G G G    
294	. A A A    
295	. G G G    
296	. G G G    
297	. A A A    
298	. G G G    
299	. G G G    
300	. G G G    
301	. C C C    
302	. G G G    
303	. C C C    
304	. T T T    
305	. G G G    
306	. T T T    
307	. T T T    
308	. A A A    
309	. C C C    
310	. A A A    
311	. G G G    
312	. C C C    
313	. C C C    
314	. C C C    
315	. C C C    
316	. C C C    
317	. T T T    
318	. G G G    
319	. T T T    
320	. C C C    
321	. T T T    
322	. G G G    
323	. T T T    
324	. C C C    
325	. T T T    
326	. T T T    
327	. C C C    
328	. C C C    
329	. A A A    
330	. G G G    
331	A A A A    
332	C C C C    
333	C C C C    
334	C C C C    
335	C C C C    
336	T T T T    
337	C C C C    
338	A A A A    
339	A A A A    
340	C C C C    
341	C C C C    
342	A A A A    
343	A A A A    
344	T T T T    
345	C C C C    
346	A A A A    
347	A A A A    
348	A A A A    
349	G G G G    
350	A A A A    
351	G G G G    
352	G G G G    
353	A A A A    
354	G G G G    
355	G G G G    
356	A A A A    
357	G G G G    
358	G G G G    
359	C C C C    
360	C C C C    
361	G G G G    
362	A A A A    
363	G G G G    
364	C C C C    
365	C C C C    
366	T T T T    
367	C C C C    
368	C C C C    
369	A A A A    
370	G G G G    
371	C C C C    
372	G G G G    
373	C C C C    
374	A A A A    
375	G G G G    
376	A A A A    
377	C C C C    
378	A A A A    
379	G G G G    
380	A A A A    
381	C C C C    
382	A A A A    
383	C C C C    
384	A A A A    
385	C C C C    
386	G G G G    
387	G G G G    
388	G G G G    
389	A A A A    
390	C C C C    
391	T T T T    
392	T T T T    
393	G G G G    
394	A A A A    
395	A A A A    
396	A A A A    
397	A A A A    
398	G G G G    
399	G G G G    
400	T T T T    
401	A A A A    
402	T T T T    
403	G G G G    
404	A A A A    
405	G G G G    
406	A A A A    
407	A A A A    
408	G G G G    
409	G G G G    
410	G G G G    
411	T T T T    
412	G G G G    
413	G G G G    
414	T T T T    
415	G G G G    
416	G G G G    
417	T T T T    
418	T T T T    
419	C C C C    
420	C C C C    
421	A A A A    
422	G G G G    
423	T T T T    
424	C C C C    
425	T T T T    
426	G G G G    
427	G G G G    
428	A A A A    
429	G G G G    
430	G G G G    
431	G G G G    
432	T T T T    
433	G G G G    
434	A A A A    
435	A A A A    
436	G G G G    
437	G G G G    
438	C C C C    
439	C C C C    
440	C C C C    
441	C C C C    
442	A A A A    
443	C C C C    
444	C C C C    
445	C C C C    
446	C C C C    
447	A A A A    
448	C C C C    
449	A A A A    
450	C C C C    
451	A A A A    
452	G G G G    
453	. C C C    
454	. C C C    
455	. A A A    
456	. A A A    
457	. C C C    
458	. C C C    
459	. A A A    
460	. G G G    
461	. C C C    
462	. A A A    
463	. C C C    
464	. A A A    
465	. G G G    
466	. C C C    
467	. T T T    
468	. G G G    
469	. T T T    
470	. T T T    
471	. C C C    
472	. T T T    
473	. T T T    
474	. C C C    
475	. A A A    
476	. C C C    
477	. C C C    
478	. C C C    
479	. C C C    
480	. T T T    
481	. G G G    
482	. A A A    
483	. A A A    
484	. C C C    
485	. C C C    
486	. C C C    
487	. A A A    
488	. G G G    
489	. C C C    
490	. C C C    
491	. T T T    
492	. G G G    
493	. A A A    
494	. G G G    
495	. G G G    
496	. A A A    
497	. G G G    
498	. G G G    
499	. G G G    
500	. C C C    
501	. C C C    
502	. A A A    
503	. A A A    
504	. T T T    
505	. G G G    
506	. A A A    
507	. C C C    
508	. A A A    
509	. T T T    
510	. G G G    
511	. C C C    
512	. A A A    
513	. G G G    
514	. G G G    
515	. C C C    
516	. C C C    
517	. C C C    
518	. A A A    
519	. A A A    
520	. C C C    
521	. T T T    
522	. G G G    
523	. C C C    
524	. G G G    
525	. C C C    
526	. T T T    
527	. A A A    
528	. C C C    
529	. A A A    
530	. A A A    
531	. C C C    
532	. A A A    
533	. C C C    
534	. A A A    
535	. C C C    
536	. C C C    
537	. C C C    
538	. A A A    
539	. T T T    
540	. C C C    
541	. A A A    
542	. A A A    
543	. T T T    
544	. G G G    
545	. G G G    
546	. T T T    
547	. G G G    
548	. G G G    
549	. T T T    
550	. C C C    
551	. T T T    
552	. G G G    
553	. C C C    
554	. C C C    
555	. C C C    
556	. A A A    
557	. C C C    
558	. T T T    
559	. A A A    
560	. C C C    
561	. T T T    
562	. G G G    
563	. C C C    
564	. A A A    
565	. C C C    
566	. C C C    
567	. A A A    
568	. G G G    
569	. - - C  1	 indel  
570	. - - A  1	 indel  
571	. - - G  1	 indel  
572	. G G G    
573	. C C C    
574	. C C C    
575	. C C C    
576	. T T T    
577	. G G G    
578	. C C C    
579	. A A A    
580	. A G G  1	 transition  
581	. . T T    
582	. . T T    
583	. . T T    
584	. . C C    
585	. . T T    
586	. . T T    
587	. . C C    
588	. . A A    
589	. . G G    
590	. . T T    
591	. . G G    
592	. . G G    
593	. . T T    
594	. . A A    
595	. . G G    
596	. . C C    
597	. . A A    
598	. . T T    
599	. . C C    
600	. . C C    
601	. . T T    
602	. . C C    
603	. . T T    
604	. . G G    
605	. . T T    
606	. . A A    
607	. . T T    
608	. . C C    
609	. . A A    
610	. . G G    
611	. . A A    
612	. . G G    
613	. . A A    
614	. . G G    
615	. . A A    
616	. . A A    
617	. . T T    
618	. . A A    
619	. . G G    
620	. . T T    
621	. . G G    
622	. . G G    
623	. . A A    
624	. . T T    
625	. . G G    
626	. . C C    
627	. . T T    
628	. . G G    
629	. . T T    
630	. . T T    
631	. . G G    
632	. . G G    
633	. . C C    
634	. . G G    
635	. . C C    
636	. . A A    
637	. . G G    
638	. . A A    
639	. . G G    
640	. . G G    
641	. . G G    
642	. . T T    
643	. . G G    
644	. . G G    
645	. . A A    
646	. . G G    
647	. . G G    
648	. . C C    
649	. . A A    
650	. . C C    
651	. . T T    
652	. . C C    
653	. . C C    
654	. . C C    
655	. . C C    
656	. . T T    
657	. . C C    
658	. . A A    
659	. . C C    
660	. . A A    
661	. . T T    
662	. . C C    
663	. . C C    
664	. . C C    
665	. . T T    
666	. . T T    
667	. . C C    
668	. . A A    
669	. . G G    
670	. . A A    
671	. . T T    
672	. . C C    
673	. . C C    
674	. . A A    
675	. . G G    
676	. . T T    
677	. . T T    
678	. . C C    
679	. . A A    
680	. . T T    
681	. . C C    
682	. . C C    
683	. . G G    
684	. . C C    
685	. . A A    
686	. . A A    
687	. . C C    
688	. . A A    
689	. . T T    
690	. . G G    
691	. . A A    
692	. . T T    
693	. . C C    
694	. . C C    
695	. . A A    
696	. . C C    
697	. . G G    
698	. . A A    
699	. . G G    
700	. . A A    
701	. . C C    
702	. . C C    
703	. . C C    
704	. . T T    
705	. . G G    
706	. . G G    
707	. . A A    
708	. . A A    
709	. . G G    
710	. . A A    
711	. . C C    
712	. . T T    
713	. . T T    
714	. . C C    
715	. . A A    
716	. . G G    
717	. . G G    
718	. . G G    
719	. . A A    
720	. . C C    
721	. . G G    
722	. . C C    
723	. . C C    
724	. . T T    
725	. . G G    
726	. . C C    
727	. . C C    
728	. . A A    
729	. . C C    
730	. . C C    
731	. . G G    
732	. . A A    
733	. . G G    
734	. . A A    
735	. . C C    
736	. . A A    
737	. . T T    
738	. . A A    
739	. . G G    
740	. . T T    
741	. . G G    
742	. . A A    
743	. . A A    
744	. . C C    
745	. . C C    
746	. . T T    
747	. . C C    
748	. . C C    
749	. . A A    
750	. . G G    
751	. . G G    
752	. . T T    
753	. . G G    
754	. . G G    
755	. . A A    
756	. . G G    
757	. . A A    
758	. . T T    
759	. . G G    
760	. . G G    
761	. . T T    
762	. . C C    
763	. . A A    
764	. . A A    
765	. . A A    
766	. . C C    
767	. . A A    
768	. . G G    
769	. . T T    
770	. . T T    
771	. . C C    
772	. . T T    
773	. . A A    
774	. . C C    
775	. . A A    
776	. . T T    
777	. . T T    
778	. . C C    
779	. . A A    
780	. . G G    
781	. . C C    
782	. . T T    
783	. . G G    
784	. . A A    
785	. . A A    
786	. . C C    
787	. . G G    
788	. . A A    
789	. . G G    
790	. . A A    
791	. . T T    
792	. . C C    
793	. . C C    
794	. . A A    
795	. . T T    
796	. . G G    
797	. . G G    
798	. . T T    
799	. . C C    
800	. . T T    
801	. . G G    
802	. . A A    
803	. . T T    
804	. . C .    
805	. . G .    
806	. . A .    
807	. . G .    
808	. . A .    
809	. . A .    
810	. . A .    
811	. . T .    
812	. . A .    
813	. . C .    
814	. . T .    
815	. . C .    
816	. . A .    
817	. . G .    
818	. . T .    
819	. . C .    
820	. . A .    
821	. . A .    
822	. . C .    
823	. . G .    
824	. . A .    
825	. . C .    
826	. . A .    
827	. . C .    
828	. . G .    
829	. . C .    
830	. . T .    
831	. . G .    
832	. . G .    
833	. . T .
```
